# In Vitro Conditions Research of *Sophora koreensis* Nakai for Shoot Elongation

**DOI:** 10.3390/plants14111692

**Published:** 2025-05-31

**Authors:** Hwa Lee, Gyu Il Han, Kyeong-Seong Cheon, Eun Ju Cheong

**Affiliations:** 1 Division of Tree Improvement and Biotechnology, Department of Forest Bio-Resources, National Institute of Forest Science, Suwon 16631, Republic of Korea; dlghk018@kangwon.ac.kr; 2 Division of Forest Conservation and Restoration, Baekdudaegan National Arboretum, Bonghwa 36209, Republic of Korea; gyu1@koagi.or.kr; 3 Division of Forest Science, College of Forest and Environmental Sciences, Kangwon National University, Chuncheon 24341, Republic of Korea

**Keywords:** endemic plant, micropropagation, plant growth regulator, photoperiod, subculture

## Abstract

*Sophora koreensis* Nakai, listed as endangered on the IUCN Red List, is a species native to Korea, specifically found in parts of Gangwon-do. Recent research highlights its potential in hangover relief and as an antioxidant source, sparking interest in enhancing its components through mutation for commercial purposes. Given its limited distribution, micropropagation of *S. koreensis* is essential for its economic exploitation. This study focuses on in vitro culture to develop an elongation system for micropropagation. The hormonal combination of 6-benzylaminopurine (2 μM), thidiazuron (2 μM), and indole-3-butyric acid (0.5 μM) produced the highest number of shoots (14) with an average length of 0.7 cm compared to the control. Additionally, adjusting photoperiod conditions under specific culture media further increased shoot length to 0.6 cm, which was also higher than that of the corresponding control group under standard light conditions. However, survival rates were generally low across all treatments during subculture. Isolating and individually culturing induced explants resulted in shorter shoots and lower survival rates. Improvements were noted when explants with 10 shoots were subcultured, achieving an 83% survival rate, with an average of 4.93 shoots at 0.95 cm in length. Rooting was most successful with 10 μM IBA, also showing the highest root number, indicating a potential pathway for enhancing micropropagation efficiency.

## 1. Introduction

*Sophora koreensis Sophora koreensis* is a member of the Fabaceae family, commonly known as Korean Sophora, and is native to the Korean peninsula. It is found in various habitats, including mountainous regions and forests, but its distribution is restricted to Gangwon-do province, such as Chuncheon, Inje, and Yanggu, Korea. It is a deciduous shrub that grows to about 1 m in height and expands its distribution more by root suckers than by seeds [1]. It has been known as a monotypic and endemic genus of Papilionoideae of Fabaceae in Korea. However, it was controversial whether it is distinct from or merged with *Sophora* [2]. Although it was named *Sophora* or *Echinosophora* in some research, we will use ‘*Sophora*’ according to [3] because phylogenetic analysis suggested that *S. koreensis* is closely related to the genus *Sophora alopecuroides* var. *alopecuroides* within Fabaceae.

*S. koreensis* is a native Korean species valued for its ornamental, ecological, and medicinal properties. Its delicate foliage and bright yellow flowers make it popular in landscaping and ecological restoration due to its ability to thrive under harsh environmental conditions. Additionally, *S. koreensis* has been traditionally used in Korean medicine for treating gastrointestinal disorders [4], and recent studies have reported its anti-inflammatory, anti-tumor, and antiviral properties [5,6]. These pharmacological potentials have drawn attention to the plant as a promising candidate for developing novel medicinal compounds [7].

The utilization of indigenous plants such as *S. koreensis* in the bio-industry also aligns with the principles of the Nagoya Protocol, which emphasizes the fair and equitable sharing of benefits arising from the use of genetic resources. However, the natural habitats of *S. koreensis* have been severely impacted by urban development and agricultural expansion. As a result, it has been classified as an endangered species by the IUCN Red List due to habitat loss and fragmentation. To prevent its extinction and ensure sustainable use, it is crucial to establish effective methods for propagation and conservation. In vitro culture techniques provide a viable strategy to produce uniform, disease-free plant material while enabling the production of bioactive compounds under controlled conditions [8,9,10].

Although recent studies have explored in vitro propagation for medicinal plants [11], protocols specific to *S. koreensis* remain underdeveloped. A few reports have employed in vitro culture techniques to propagate *S. koreensis* using shoots with axillary buds [12].

However, these studies often encountered limitations in shoot elongation, which is critical for successful acclimatization and transplantation. In plant tissue culture, both shoot multiplication and elongation are essential for producing viable plantlets for conservation and industrial use. Therefore, this study aims to establish an efficient in vitro propagation system for *S. koreensis*. By identifying the factors that limit shoot elongation and optimizing culture conditions, we seek to enhance the effectiveness of in vitro propagation. This approach will support the sustainable conservation of this endangered species and promote its practical application in the bio-industry.

## 2. Results

### 2.1. Effects of Growth Regulator Combinations on Shoot Multiplication

In this experiment, various hormone treatments were applied. The combination of BA(6-benzylaminopurine) with TDZ(thidiazuron) produced approximately threefold more shoots compared to treatment with BA alone (Table 1), indicating that the addition of TDZ was more effective for shoot multiplication. A concentration of 2.27 µM of TDZ produced an average of 3.15 shoots [12]. GA_3_ was more effective in promoting shoot multiplication rather than elongation. The highest number of shoots, averaging 11.30, was observed when GA_3_ (Gibberellic Acid) was applied at 10 µM.

When a low concentration of auxin, particularly NAA(α-Naphthaleneacetic Acid), was applied, an increase in the number of shoots was observed in *S. koreensis*. In contrast, the addition of IBA(indole-3-butyric acid) promoted shoot length. Although the average shoot length with IBA treatment was similar to that observed with BA alone, IBA induced approximately 15 times more shoots. However, higher concentrations of IBA had a negative effect on shoot elongation.

### 2.2. Shoot Elongation as Affected by Environmental and Cultural

#### 2.2.1. Photoperiod

**Figure 1:**
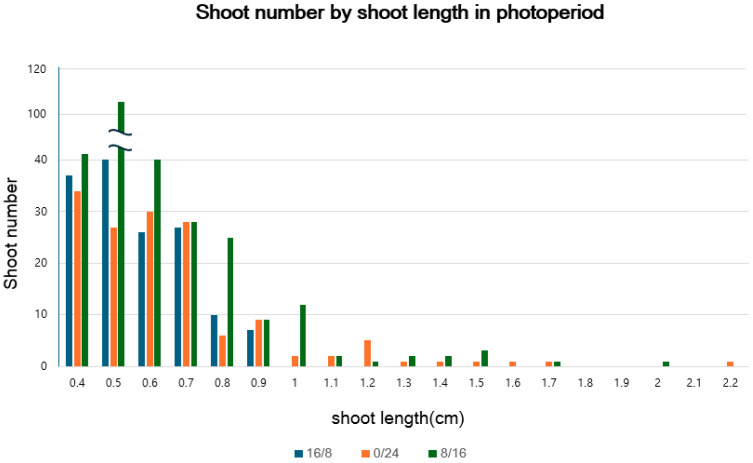
Shoot length frequency under three light–dark regimes.

**Figure 2:**
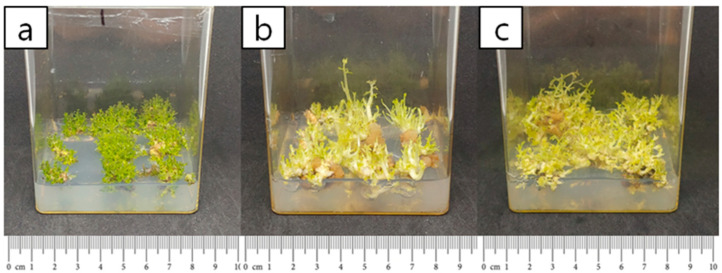
Effect of photoperiod on shoot length: (**a**) 16 h light/8 h dark, (**b**) continuous darkness, (**c**) 8 h light/16 h dark.

The average shoot length under the 8/16 (light/dark) photoperiod was the highest at 0.60 cm, with a statistically significant difference (Table 2). There was no statistically significant difference between darkness and the 16/8 photoperiod under fluorescent light. This is because newly generated shoots were short in length but numerous, making it difficult to detect significant differences when analyzing the average. However, naked-eye observations confirmed that the shoots of plants grown under darkness (0/24 photoperiod) were much longer, with the longest shoot length reaching up to 2.2 cm, as shown in Table 2. The number of shoots for each treatment was plotted on a graph based on their respective lengths (Figure 1). When comparing the 16/8 photoperiod with continuous darkness, although the average shoot length was not the highest under the dark treatment (Table 2), the proportion of longer shoots (exceeding 0.6 cm) was relatively greater, as shown in Figure 1. Overall, the fluorescent 8/16 photoperiod, which resulted in the longest average shoot length among the three light treatments, was the most optimal condition for shoot elongation in *S. koreensis*. However, when considering the number of shoots within each length category, darkness was more effective in generating longer shoots compared to the 16/8 fluorescent light treatment. As the duration of darkness increased, etiolation of the cultures was observed (Figure 2).

#### 2.2.2. Subculture

In this study, the initial culture in MS medium supplemented with 2 µM BA, 2 µM TDZ, and 1 µM GA_3_ exhibited the highest average survival rate of 77% (Table 3). Additionally, it showed the longest average shoot length of 0.77 cm, with a significant difference.

The addition of GA_3_ in the initial culture medium resulted in more vigorous shoot elongation after subculture. When subcultured under the same conditions (MS medium with 2 µM BA, 2 µM TDZ, and 1 µM GA_3_), the survival rate was highest at 90%, and the shoot length was also the longest at 0.91 ± 0.16 cm.

**Figure 3:**
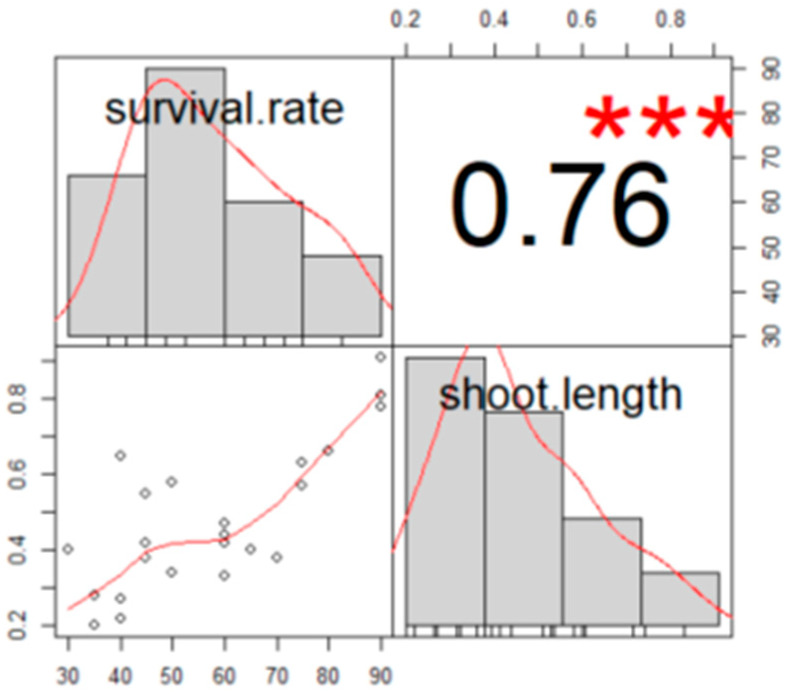
Correlation between survival rate (%) and shoot length (cm). Pearson correlation coefficients, and significance is denoted by asterisks (*** *p* ≤ 0.001).

Analysis of the correlation between survival rate and average shoot length indicated a positive correlation, with a correlation coefficient of 0.756 (Figure 3). This suggests that longer average shoot length is associated with higher survival rates.

The inclusion of GA_3_ in the initial culture medium resulted in better elongation growth, which is attributed to varying response times to absorbed GA_3_ depending on species. It is believed that for *S. koreensis*, a longer observation period beyond the initial 4 weeks may be necessary to fully observe the elongation effects of GA_3_.

#### 2.2.3. The Effect of Shoot Cluster Size in Subculture

**Figure 4:**
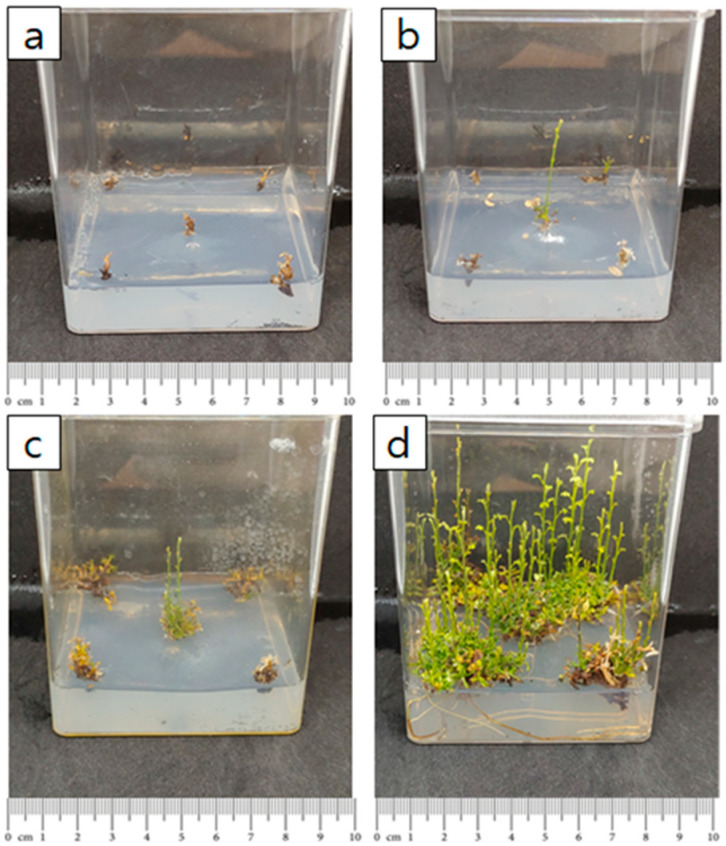
Effect of initial explant clump size on *S. koreensis* cultures: (**a**) one shoot, (**b**) two shoots, (**c**) five shoots, (**d**) ten shoots.

**Figure 5:**
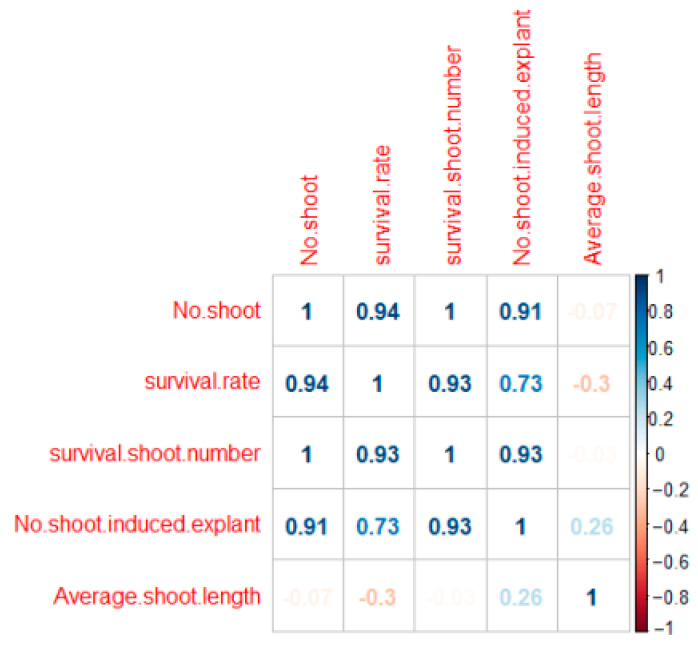
Correlation shoot growth of *S. koreensis* and explant number.

As the number of shoots increased, the survival rate of subcultured explants increased (Table 4). The highest survival rate, 83%, was observed when 10 shoots were subcultured. In this treatment, the average number of shoots was about 5, and the average shoot length was 0.95 cm, showing a significant difference from other treatments. Although the survival rate and shoot length were lowest when a single shoot was subcultured, the number of shoots generated still increased compared to the initial culture. In contrast, subculturing with 2, 5, and 10 explants resulted in a reduced number of newly generated shoots compared to the number of shoots initially cultured. Subculturing explants with 10 shoots produced approximately 5 shoots with an average length of 0.95 cm. Therefore, utilizing explants with 10 shoots during culture appears to be the most effective approach for promoting shoot elongation (Figure 4). Correlation analysis between the number of explants and survival rate revealed a strong positive correlation, with a correlation coefficient of 0.9 or higher (Figure 5).

### 2.3. Effect of Auxin Treatments on Root Induction

The results of the experiment to determine the rooting induction conditions for *S. koreensis* are presented in Table 5 below. This experiment evaluated the effects of treating shoots with the auxins IBA and NAA individually for rooting induction.

**Figure 6:**
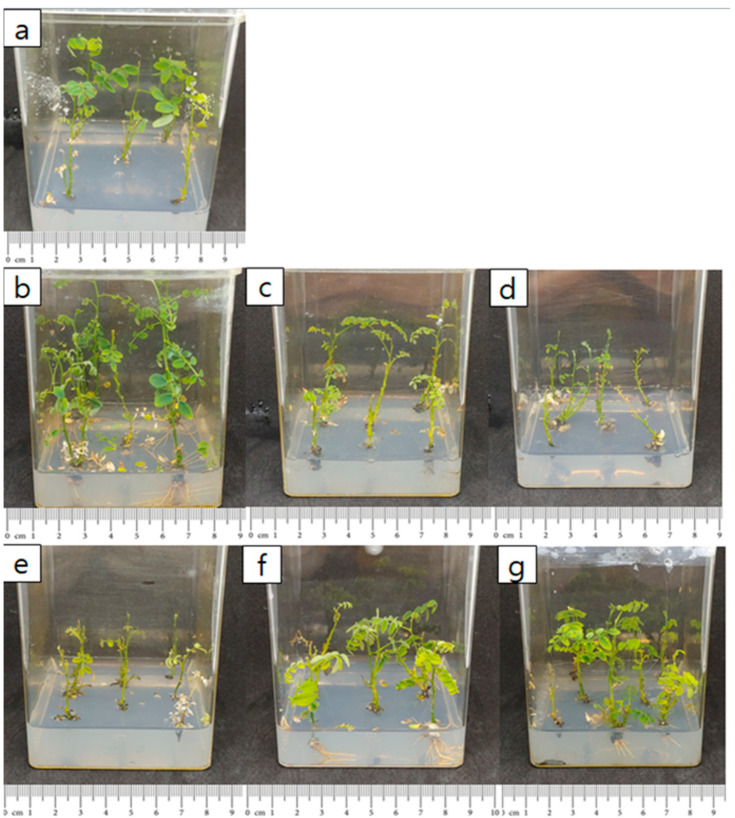
Rooting comparison of *S. koreensis* according to hormone concentration. (**a**) Control; (**b**) NAA 1 µM; (**c**) NAA 5 µM; (**d**) NAA 10 µM; (**e**) IBA 1 µM; (**f**) IBA 5 µM; (**g**) IBA 10 µM.

In the absence of auxins, the rooting rate was 4%, the lowest among all treatment groups. Treatment with IBA resulted in an increased rooting rate with rising concentrations, ranging from 8% up to a maximum of 60% at 10 µM IBA. Similarly, NAA treatment improved rooting rates from 16% to a maximum of 32% at 10 µM NAA. Consequently, 10 µM IBA proved to be the optimal rooting condition, displaying the highest rooting rate and greatest root induction. The average root length showed no significant differences, indicating that neither NAA nor IBA treatments substantially affected root length. Although root development is not clearly visible in the current images, visual observations during the experiment confirmed that plantlets treated with 10 µM IBA developed white, well-branched roots, demonstrating successful rooting under optimal induction conditions (Figure 6).

### 2.4. Acclimatization in Soil

**Figure 7:**
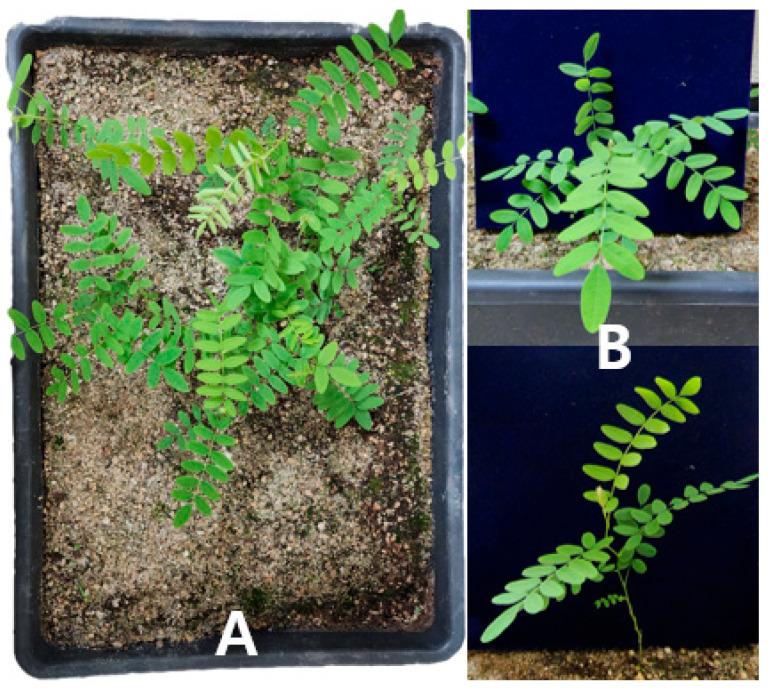
Acclimatized *S. koreensis* plantlets six weeks after transfer to soil. (**A**) Overview of multiple plantlets in a tray under greenhouse conditions. (**B**) Close-up view showing upright shoot growth and leaf development.

After six weeks of acclimatization, 97 out of 127 *Sophora koreensis* plantlets survived, resulting in a survival rate of 76.3%. During this period, the plantlets continued to grow, reaching an average shoot length of 11.8 cm under greenhouse conditions (Figure 7).

## 3. Discussion

When BA, a cytokinin, was added to the medium, the survival rate increased and two stems were generated. Cytokinins have been shown to be critical growth regulators for shoot elongation in many medicinal plant species [13,14,15,16]. Additionally, treatment with TDZ produced six stems, a threefold increase. TDZ is known to promote meristem formation and new shoot proliferation, especially at low concentrations. Treatment with 2.27 µM TDZ produced an average of 3.15 stems, but these were observed to be short in length [12]. The combined use of two or more plant growth regulators during in vitro culture has been reported to enhance multiplication rates [17]. This aligns with the known role of cytokinins in promoting stem proliferation while inhibiting elongation growth [18]. Similarly, *Sophora tonkinensis*, a member of the same genus, showed effective induction of multiple shoots when treated with the cytokinin 2iP, although shoot length was not significantly increased [19].

At the combination of TDZ and BA, the regenerated shoots were short and slender, exhibited abnormal leaf morphology, and showed adventitious shoots at the basal and nodal regions of multiple shoots. In this case, there was likely an excess of TDZ and BA competing for binding sites, making them toxic to plant cells. These results are in agreement with observations reported by other investigators [20,21,22,23].

A previous study [24] demonstrated that GA_3_ can enhance plant growth. These data show that GA_3_ is beneficial for improving biomass production. This effect may be explained by GA_3_’s known role in promoting cell elongation, as observed by Davidonis [25] in cotton cells. One study showed that BA treatment, including GA_3_, was not involved in stem induction of *S. koreensis* [12]. In contrast, the findings of this study indicate that the addition of TDZ tends to increase shoot numbers when combined with higher concentrations of GA_3_.

It has been reported that the addition of a low concentration of auxin results in increased shoot induction and elongation [26]. A higher ratio of cytokinin to auxin (0.1 mg L^−1^ NAA and 0.6 mg L^−1^ BA) was used in the medium for shoot multiplication [27]. Overall, this aligns with the concept that appropriate ratios of cytokinins and auxins—particularly higher concentrations of cytokinin relative to auxin—in the shoot induction medium favor shoot formation [28].

The addition of GA_3_ in the initial culture medium resulted in more vigorous shoot elongation after subculture. However, it is believed that when GA_3_ is included at too high a concentration (10 µM), the effect becomes adverse. In a preliminary study, it was also observed that growth decreased when BA and GA_3_ were applied together and GA_3_ exceeded a certain concentration [29].

Among environmental factors affecting plant growth, photoperiod is one of the most critical, regulating growth by maintaining a specific ratio of photoreceptors during particular periods [30,31,32]. When morphological characteristics were examined by controlling the photoperiod, a higher ratio of darkness resulted in increased shoot length [33]. In this study, the fluorescent 8/16 (light/dark) photoperiod was determined to be the most optimal condition for shoot length development in *S. koreensis*. Although increased darkness led to longer stems, etiolation was also observed. Shoots developed in the absence of light exhibit elongated buds and expanded nodes, displaying typical characteristics of etiolated growth. When a seedling emerges from the soil into sunlight, stem elongation is suppressed, and development shifts from vertical growth to the expansion of photosynthetic capacity. Both the blue and red components of white light contribute to the inhibition of stem elongation [34].

The study found that the initial culture medium supplemented with 2 μM BA, 2 μM TDZ, and 1 μM GA_3_ exhibited the highest average survival rate (77%) and longest average shoot length (0.77 cm) for *S. koreensis* and that the addition of GA_3_ during subculture further enhanced shoot elongation. Previous studies have investigated the effects of hormones on plant regeneration during subculture [35], as well as the impact of subculture intervals [36]. However, reports on the effect of the initial culture medium on subculture are scarce. This may be because the hormone concentrations in the initial culture medium were too high, hindering growth initially, with hormone effects appearing later in lower concentration media.

In this experiment, the overall survival rate was lower compared to other studies, which is attributed to the difficulty in acclimatization, likely due to culturing one shoot per explant during subculture.

Although few experiments have focused on controlling the number of shoots per explant, this approach could be applicable in cases like *S. koreensis*, where survival rates are low when culturing a single shoot. In plant tissue culture, plantlets grown in clusters often exhibit higher survival rates than individual plantlets. Cluster cultivation allows more efficient utilization of resources such as nutrients, water, and light, which can be shared among plantlets rather than provided individually. This creates a more favorable growth environment and increases survival.

Moreover, interdependence among clustered plantlets offers mutual physical support, reducing the risk of damage and thereby enhancing overall survival rates [37]. Cluster cultivation can also mitigate stress experienced by individual plantlets during tissue culture, as plantlets benefit from the presence of neighbors and may be less affected by environmental fluctuations such as temperature or humidity changes [38].

Therefore, cluster cultivation in tissue culture can be a valuable strategy to improve plantlet survival and growth. By leveraging mutual support and stress reduction, researchers and growers can optimize tissue culture outcomes, particularly under challenging environmental conditions or when individual plantlets are vulnerable to damage.

There have been many studies on the influence of auxins on adventitious root formation [39,40]. It has been reported that the differentiation of plant organs is influenced by the ratio of auxins to cytokinins in the medium, with a higher proportion of auxins promoting the formation of adventitious roots. Within the genus *Sophora*, *Sophora flavescens* exhibited an average rooting rate of 82.4% on a medium containing high concentrations of BA and low concentrations of NAA [41].

In a previous study, rooting experiments conducted over 8 weeks showed that the rooting rate in the IBA treatment group increased up to 10%, while in the NAA treatment group, it increased from 40% to 62.5% [12]. These differences may be attributed to species-specific physiological and ecological characteristics, as well as the relatively short experimental duration of 4 weeks. However, in our study, similar root induction rates were achieved in only half the time of the previous study. Root induction was most prominent in treatments with 10 µM IBA and 1 µM NAA. Consistent with prior observations [12], higher concentrations of IBA induced a greater number of roots.

The observed survival rate of 76.3% indicates that in vitro regenerated *S. koreensis* plantlets exhibited moderate adaptability to ex vitro conditions. Although no quantitative measurements of morphological traits were taken, the plantlets appeared visually healthy, with upright stems and well-formed green leaves. Previous studies have emphasized the role of initial shoot development in acclimatization success. For instance, García-Ramírez et al. [42] reported that plantlets of *G. aff. chaparensis* with more than two elongated shoots showed significantly higher survival rates, while those with shorter or fewer shoots were more vulnerable during the ex vitro transition.

In our study, an average shoot length of 11.8 cm after six weeks of acclimatization suggests that pre-acclimatization elongation contributed positively to survival. Longer shoots are likely to enhance structural stability and photosynthetic activity during the early stages of soil establishment. These findings highlight the importance of optimizing elongation conditions during in vitro culture to ensure successful acclimatization.

Future studies should incorporate quantitative evaluations of branching, root development, and leaf morphology to better understand the traits influencing ex vitro survival.

## 4. Materials and Methods

### 4.1. Plant Materials and Culture Conditions

Shoots were collected from Chuncheon-si, Gangwon-do, Korea. The collected shoots were segmented into nodal explants containing 1–2 axillary buds. The explants were initially sterilized in a solution containing one drop of Tween 40 for approximately 10 min. This was followed by disinfection in an ultrasonic cleaner for 20 min. Subsequently, the explants were shaken at 120 rpm for 15–20 min in a 1% (*v*/*v*) sodium hypochlorite (NaOCl) solution. After surface sterilization, the explants were rinsed more than five times with sterile distilled water under a laminar flow hood. The disinfected nodal segments were then used as explants. For culture, MS medium supplemented with 3% sucrose, BA 2 µM, 4.5 g/L agar, and 1.25 g/L Gelrite was used. The pH of the medium was adjusted to 5.8 before autoclaving. The shoots regenerated from this medium were used for further experiments. For all experiments, in vitro shoots derived from axillary buds were used. Culture medium was based on the MS (Murashige and Skoog) supplemented with 3% sucrose and adjusted to pH 5.8. Twenty mL of culture medium were dispensed in the magenta box and solidified with 7 g/L of agar, then sterilized by autoclaving at 121 °C and 15~20 psi for 20 min. Cultures were maintained at 24 ± 1 °C, and light was provided with white fluorescent lamps. The photosynthetic photon flux density (PPFD) of the light source was set at 70 µmol·m^−2^·s^−1^. Ten explants per treatment were tested, and the experiment was repeated three times.

### 4.2. Synergistic Effect of Gibberellic Acid and Auxins with Cytokinins for Shoot Multiplication

Four different concentrations of GA_3_ (1, 2, 5, and 10 µM) and three concentrations of NAA and IBA (0.5, 1, 2 and µM) were supplemented in the basic culture medium, which has two cytokinins (2 µM of BA and TDZ). Hormone-free and BA-only medium were used as a control. The survival rate (%) of the explants, the number of induced shoots per explant, and the average shoot length were measured within four weeks of culture.

### 4.3. Factors Influencing Shoot Elongation

#### 4.3.1. Gibberellic Acid in the Subsequent Culture Medium for Shoot Growth

To investigate the effect of the subsequent culture medium on shoot growth, especially shoot elongation, and the accumulative effect of the previous culture medium, shoots grown on different culture media were subcultured to the medium containing 1, 2, and 5 µM of GA_3_. The survival rate (%) of the explants, the number of induced shoots per explant, and the average shoot length were measured within four weeks of culture.

#### 4.3.2. Photoperiod on Shoot Growth

Shoots measuring at least 0.4 cm in length were used as explants for culture. These shoots were placed under three different light conditions: 16 h light/8 h dark, 8 h light/16 h dark, and continuous dark. Four weeks later, the number of shoots induced, and length were measured.

#### 4.3.3. Shoot Growth on Shoot Clump Numbers

Shoots induced from the primary culture were divided into various sizes in micro-shoot clumps. Some were excised individually and placed on the medium, and others consisted of 2, 5, and 10 micro shoots. We examined the survival rate and shoot elongation by the shoot clump numbers.

### 4.4. Root Induction by Auxin Treatments

Elongated shoots were placed in a culture medium supplemented with 1, 5, and 10 µM of IBA or NAA. No hormones were contained in the medium as a control. Rooting parameters, including rooting percentage, number of roots per shoot, and average root length, were recorded after 6 weeks of culture.

### 4.5. Soil Acclimatization

To acclimatize the in vitro cultures, two root-induced plants were transplanted into a soil mixture of sand and bed soil in a 7:3 ratio. The plants were kept at a temperature of 24 ± 1 °C with a 16 h light and 8 h dark photoperiod provided by white fluorescent lamps. Humidity was maintained at 60%. The survival rate was assessed six weeks after transplantation into the soil. During soil acclimatization, plantlets were maintained under a moderate light intensity of approximately 120 µmol·m^−2^·s^−1^ PPFD to facilitate gradual adaptation to ex vitro conditions. The plantlets were irrigated once daily with tap water to maintain adequate soil moisture.

### 4.6. Data Analysis

Statistical analysis was performed by two-way ANOVA using R software (R version 4.0.3, GNU General Public License, New Zealand). Tukey’s test was performed as a post hoc analysis to test for differences in means between groups, and basic statistics were calculated and homogeneous subsets were derived for each hormone treatment.

## 5. Conclusions

In this study, we established a reliable in vitro propagation protocol for *Sophora koreensis*, addressing previously reported limitations in shoot survival and elongation. This system not only supports ex situ conservation of this rare Korean endemic species but also lays the foundation for potential commercial applications through stable production of plant material. Future research will focus on enhancing secondary metabolite accumulation, improving rooting and acclimatization, and ensuring genetic stability to broaden both conservation and industrial utilization.

## Figures and Tables

**Table 1:** Shoot production of *S. koreensis* according to hormone and concentration.

Treatment (µM)	Survival Rate (%)	No. of Shoot Induced/Explant	Average Shoot Length (cm)
Control	90	3.70 ± 0.76 ^ab^*	0.47 ± 0.05 ^a^
MS B2	100	2.37 ± 0.29 ^a^	0.70 ± 0.09 ^b^
MS B2T2	100	6.97 ± 0.50 ^bc^	0.57 ± 0.03 ^ab^
MS B2T2G1	100	6.93 ± 0.82 ^bc^	0.57 ± 0.03 ^ab^
MS B2T2G2	100	8.97 ± 0.78 ^cd^	0.52 ± 0.04 ^a^
MS B2T2G5	100	9.27 ± 0.40 ^cd^	0.52 ± 0.03 ^a^
MS B2T2G10	100	11.30 ± 0.87 ^de^	0.50 ± 0.02 ^a^
MS B2T2NAA0.5	100	16.70 ± 1.43 ^fg^	0.47 ± 0.01 ^a^
MS B2T2NAA1	100	18.37 ± 1.41 ^g^	0.50 ± 0.01 ^a^
MS B2T2NAA2	100	15.33 ± 1.00 ^efg^	0.54 ± 0.02 ^ab^
MS B2T2IBA0.5	100	13.80 ± 1.00 ^efg^	0.70 ± 0.05 ^b^
MS B2T2IBA1	100	15.40 ± 1.26 ^fg^	0.59 ± 0.02 ^ab^
MS B2T2IBA2	100	14.23 ± 1.43 ^efg^	0.50 ± 0.02 ^a^

* The different letters in the column indicate a significant difference at *p* < 0.05 by the Tukey HSD test. B: 6-Benzylaminopurine, T: Thidiazuron, G: Gibberellic acid 3.

**Table 2:** Shoot number and length of *S. koreensis* according to light conditions.

Photoperiod (Light/Dark h)	No. of Shoot Induced/Explant	Average Shoot Length(cm)	Longest Shoot Length(cm)
Light (16/8)	8.40 ± 1.01	0.46 ± 0.02 ^a^*	0.9
Darkness (0/24)	8.60 ± 0.95	0.49 ± 0.02 ^a^	2.2
Light (8/16)	8.90 ± 0.88	0.60 ± 0.02 ^b^	1.1

* The different letters in the column indicate significant differences at *p* < 0.05 by the Tukey HSD test.

**Table 3:** Effects of BA–TDZ shoot initiation and GA-supplemented subculture media on survival, shoot multiplication, and elongation of *S. koreensis* explants in vitro.

Treatment (µM)		Survival Rate (%)	No. of Shoot Induced/Explant	Average Shoot Length (cm)
Shoot Initiation	Subculture Medium			
B2	MS hormone-free	70	0.70 ± 0.11 ^abc^*	0.38 ± 0.06 ^abc^
	MS G1	60	0.70 ± 0.13 ^abc^	0.33 ± 0.06 ^ab^
	MS G2	40	0.35 ± 0.11 ^a^	0.22 ± 0.06 ^a^
	MS G5	35	0.35 ± 0.11 ^a^	0.28 ± 0.10 ^ab^
		51	0.53 ± 0.06 ^a^	0.30 ± 0.04 ^a^
B2T2	MS hormone-free	35	0.55 ± 0.11 ^ab^	0.20 ± 0.06 ^a^
	MS G1	60	0.80 ± 0.12 ^abc^	0.47 ± 0.07 ^abc^
	MS G2	60	1.00 ± 0.22 ^abc^	0.44 ± 0.09 ^abc^
	MS G5	50	0.60 ± 0.17 ^ab^	0.58 ± 0.19 ^abc^
		53	0.74 ± 0.08 ^ab^	0.45 ± 0.06 ^ab^
B2T2G1	MS hormone-free	90	0.95 ± 0.11 ^abc^	0.91 ± 0.16 ^c^
	MS G1	75	0.85 ± 0.11 ^abc^	0.63 ± 0.10 ^abc^
	MS G2	90	1.25 ± 0.20 ^bc^	0.81 ± 0.10 ^bc^
	MS G5	45	0.90 ± 0.24 ^abc^	0.42 ± 0.09 ^abc^
		77	0.99 ± 0.09 ^bc^	0.77 ± 0.07 ^c^
B2T2G2	MS hormone-free	45	0.95 ± 0.18 ^abc^	0.55 ± 0.12 ^abc^
	MS G1	40	1.10 ± 0.24 ^abc^	0.65 ± 0.15 ^abc^
	MS G2	75	0.85 ± 0.11 ^abc^	0.57 ± 0.09 ^abc^
	MS G5	90	1.50 ± 0.21 ^c^	0.78 ± 0.10 ^bc^
		70	1.10 ± 0.10 ^c^	0.73 ± 0.07 ^bc^
B2T2G5	MS hormone-free	80	1.25 ± 0.19 ^bc^	0.66 ± 0.09 ^abc^
	MS G1	65	0.80 ± 0.14 ^abc^	0.40 ± 0.07 ^abc^
	MS G2	60	0.85 ± 0.18 ^abc^	0.42 ± 0.09 ^abc^
	MS G5	45	0.65 ± 0.18 ^ab^	0.38 ± 0.10 ^abc^
		62	0.89 ± 0.09 ^bc^	0.54 ± 0.06 ^ab^
B2T2G10	MS hormone-free	30	0.35 ± 0.13 ^a^	0.40 ± 0.17 ^abc^
	MS G1	40	0.40 ± 0.13 ^a^	0.27 ± 0.08 ^ab^
	MS G2	45	0.50 ± 0.11 ^ab^	0.42 ± 0.12 ^abc^
	MS G5	50	0.75 ± 0.20 ^abc^	0.34 ± 0.09 ^ab^
		40	0.50 ± 0.08 ^a^	0.37 ± 0.06 ^a^

* The different letters in a column indicate significant differences at *p* < 0.05 by the Tukey HSD test. B: 6-Benzylaminopurine, T: Thidiazuron, G: Gibberellic acid 3

**Table 4:** Influence of explant type (initial micro-shoot clump size) on survival, shoot multiplication, and elongation during in vitro subculture.

No. Shoot	Survival Rate (%)	Total Number of Induced Shoots	No. of Shoot Induced/Explant	Average Shoot Length (cm)
1	40	12	1.56 ± 0.34 ^a^*	0.39 ± 0.07 ^a^
2	50	30	1.16 ± 0.39 ^a^	0.40 ± 0.10 ^a^
5	73	110	1.56 ± 0.25 ^a^	0.53 ± 0.10 ^a^
10	83	249	4.93 ± 0.76 ^b^	0.95 ± 0.12 ^b^

* The different letters in a column indicate significant differences at *p* < 0.05 by the Tukey HSD test.

**Table 5:** Effect of IBA and NAA concentrations on in vitro rooting efficiency of *S. koreensis*.

Treatment (µM)	Rooting (%)	No. of Root Induced	Average Root Length (cm)
Control	4	0.04 ± 0.04 ^a^*	0.16 ± 0.16 ^a^
IBA 1	8	0.08 ± 0.06 ^a^	0.02 ± 0.01 ^a^
IBA 5	28	0.80 ± 0.33 ^ab^	0.12 ± 0.04 ^a^
IBA 10	60	2.04 ± 0.49 ^b^	0.16 ± 0.03 ^a^
NAA 1	24	2.00 ± 0.90 ^b^	0.14 ± 0.06 ^a^
NAA 5	16	0.28 ± 0.15 ^ab^	0.03 ± 0.01 ^a^
NAA 10	32	1.24 ± 0.49 ^ab^	0.07 ± 0.02 ^a^

* The different letters in a column indicate significant differences at *p* < 0.05 by the Tukey HSD test.

## Data Availability

All data are available within the manuscript, and further information can be obtained from the corresponding authors upon request.
